# Permanent Draft Genome Sequence of *Nocardia* sp. BMG111209, an Actinobacterium Isolated from Nodules of *Casuarina glauca*

**DOI:** 10.1128/genomeA.00770-16

**Published:** 2016-08-04

**Authors:** Faten Ghodhbane-Gtari, Nicholas Beauchemin, Abdellatif Gueddou, Karima Hezbri, Amir Ktari, Moussa Louati, Imen Nouioui, Amy Chen, Marcel Huntemann, Natalia Ivanova, Nikos Kyrpides, Victor Markowitz, Kostas Mavrommatis, Ioanna Pagani, Arnab Sen, Luis Wall, Tanja Woyke, Maher Gtari, Louis S. Tisa

**Affiliations:** a University of Tunis-El Manar, Tunis, Tunisia; b University of New Hampshire, Durham, New Hampshire, USA; c DOE Joint Genome Institute, Walnut Creek, California, USA; d University of North Bengal, Siliguri, India; e University of Quilmes, Quilmes, Argentina

## Abstract

*Nocardia* sp. strain BMG111209 is a non-*Frankia* actinobacterium isolated from root nodules of *Casuarina glauca* in Tunisia. Here, we report the 9.1-Mbp draft genome sequence of *Nocardia* sp. strain BMG111209 with a G + C content of 69.19% and 8,122 candidate protein-encoding genes.

## GENOME ANNOUNCEMENT

Actinorhizal plants are well known for their symbiotic association with the actinobacteria, *Frankia*, which results in the formation of root nodule structures (1). Besides hosting these bacterial symbionts, root nodules have other occupants or plant endophytes that seem to aid plant growth and health. Many non*-Frankia* actinobacteria have been isolated from actinorhizal root nodules, occupying the same microniche as *Frankia* (2–14). These non-*Frankia* actinobacteria appear to have functional roles including plant growth-promoting activity (4). As early as 1981, *Nocardia* species were found associated with actinorhizal nodules (7, 8). *Nocardia* sp. strain BMG111209 was isolated from the root nodules of *Casuarina glauca* grown in Tunisia (2). This isolate was sequenced to provide a greater understanding of the metabolic potential of this microbe and its interaction with actinorhizal plants.

The draft genome of *Nocardia* sp. strain BMG111209 was generated at the DOE Joint genome Institute (JGI) using a hybrid of Illumina (15) and Pacific Biosciences (PacBio) technologies. An Illumina Std. shotgun library and long insert mate-pair library were constructed and sequenced using the Illumina HiSeq 2000 platform. This procedure generated 51,070,879 reads totaling 7,660.6 Mbp from the standard shotgun library and 47,626,505 reads totaling 4,1343.5 Mb from the long insert mate-pair library. A Pacbio SMRTbell library was constructed and sequenced on the PacBio RS platform, which generated 250,559 raw PacBio reads yielding 166,907 adapter trimmed and quality filtered subreads totaling 471.4 Mb. All techniques for DNA isolation, library construction, and sequencing were performed according to JGI standards and protocols (http://www.jgi.doe.gov). The Illumina and PacBio sequence data were assembled using Allpaths–LG (version r42328) (16). The final draft assembly contained six contigs in five scaffolds. The total size of the genome is 9.1 Mbp. The final assembly is based on 7,660.9 Mbp of Illumina Standard PE, 4,143.5 Mb of Illumina CLIP and 471.4 Mb of PacBio post filtered data, which provides an average 1297.2× Illumina coverage and 51.8× PacBio coverage of the genome, respectively.

The assembled *Nocardia* BMG111209 genome was annotated using the JGI annotation pipeline (17, 18) and the data are available from the IMG data management system (19). The draft genome of *Nocardia* BMG111209 was resolved into 5 scaffolds consisting of 9,143,142 bp with a G+C content of 69.19%, 8,122 candidate protein-encoding genes, 52 tRNA genes, and 2 rRNA regions. Analysis of this genome for metabolism potential revealed phytohormone (i.e., IAA) biosynthesis pathways and the presence of a *hup* operon encoding an Ni-hydrogenase suggesting possible roles in their functioning as a plant-endophyte.

### Nucleotide sequence accession numbers.

This whole-genome shotgun sequence has been deposited at DDBJ/EMBL/GenBank under the accession number ARMU00000000. The version described in this paper is the first version, ARMU01000000.
